# The Importance of Maintaining Medical Charities

**Published:** 1886-11-13

**Authors:** 


### The Importance of Maintaining Medical Charities.

By H.R.H. The Duke of Cambridge.

Facts as to Finances.

I have the honour of being president of two great hospitals in the East of London, the London Hospital and the German Hospital. In this district we have a deficiency of £15,000, which is our proportion of the annual hospital deficiency of £40,000 or £50,000 for the whole of London. I observe from the statistics that last year no fewer than a million patients were treated in all the hospitals of London, and that the total cost was £500,000. That gives the singularly small figure of 10*s.* per patient. If we cannot keep the hospitals going a sad calamity will fall upon the community at large. The sum collected in 1885 in connection with the Metropolitan Hospital Sunday Fund was £40,000, and it consequently fell £40,000 short of the requirements of the London hospitals. This is why the large figure of £80,000 beyond the present sum raised is declared to be wanted each year for the London hospitals. The amount collected is 9*s.* per patient, and now we want to make it 10*s.* per head. These are marvellous facts. It is hardly known to the public how large an amount is expended, how small is the average expenditure on the individual patients, and how great a number of patients are represented by the additional shilling required.

The Value of Sentiment.

A leading feature in this question is that we have no State hospitals in this country, and a very good thing too, because I believe all these institutions are far better under private management by individuals who take the trouble which is taken by so many gentlemen connected with the administration of our hospitals. If we wish to keep the hospitals free from dependence upon State support, which I think is essential, these institutions must be supported by the people, not only by their sentiments, which I am sure will always go in this direction, but by positive pecuniary contributions. It is a very difficult thing to send a penny or a sixpence to a subscription list, or, it may be, to put it upon the plate on the Sunday, and to feel that one has done the right thing. You may be giving as much as you can individually, and yet feel a sense of shame at giving so little, although you have not the means to give more. It is not the amount—it is the sentiment with which it is given. Very often a penny or a sixpence represents a far finer sentiment than a £100 note. It is often a more serious thing for a poor man to give a penny than it is for a rich man to give a £100 note. That is why the Sunday collections are of such great value.

The Hospitals and Trade Depression.

There cannot be two opinions of the value and importance of such a meeting as that at Stratford in June last; and such meetings all over London each year will increase the general appreciation of the value of hospitals. Every man and woman can contribute to the great work we have in hand. When there is such a work in hand, there can be no question of convenience or inconvenience. When a man has a great object in view he sacrifices everything to attain it. No doubt many feel their pockets affected by the depressed state of trade; but sickness, disease, and accident do not stop; they do not go with the times in that respect, and the scarcity of money is no reason why there should be less sickness or fewer accidents. Nay, in times of depression sickness increases, because nourishment falls off from the want of employment, and disease resulting from want, due to non-employment, increases the demands upon the hospitals. There is, therefore, a special claim upon you to give your mite—penny, shilling, or guinea—to these great and useful institutions. If the hospitals obtain the larger sum they require, I shall feel more than ever gratified that I went to East London in the Hospitals Week, and that I have now consented to restate my views to-day on this important and pressing question.

